# A focus on chasing pharmaceutical poly*a*morphs to design better oral drug formulations

**DOI:** 10.1039/d4sc90250e

**Published:** 2025-01-27

**Authors:** Ana M. Belenguer

**Affiliations:** a Yusuf Hamied Department of Chemistry, University of Cambridge Lensfield Road Cambridge CB2 1EW UK amb84@cam.ac.uk

## Abstract

The pharmaceutical industry cares about reducing toxic side effects of drugs in oral formulation. The best solution is to reduce the drug dose. To do so, drugs are required to have high aqueous solubility to ensure good bioavailability. Amorphous drugs are much more water soluble than their crystalline counterparts, but can lack physical stability. Martins and Rades, *et al.* (I. C. B. Martins and T. Rades *et al.*, *Chem. Sci*., 2023, **14**, 11447–11455, DOI: https://doi.org/10.1039/D3SC02802J) demonstrate for the first time that poly*a*morphs (amorphous polymorphs) of drugs are now a reality. They demonstrated proof-of-concept, reproducible preparation methods for 3 poly*a*morphs (I, II and III) of hydrochlorothiazide (HCT) that display different glass transitions temperatures (*T*_g_) and distinct structural relaxation profiles as excellent analytical indicators for discriminating between the poly*a*morphs. HCT poly*a*morph-II displayed improved physical stability with respect to the other HCT poly*a*morphs. A tangible benefit of poly*a*morphism research is the opportunity to select a specific poly*a*morph of a drug with the desired solubility and physical stability to be incorporated in an oral formulation, a strategy that should improve drug effectiveness.

## Abstract 2

Martins *et al.* demonstrate how the ‘structure’ and physicochemical properties of differently prepared amorphous forms of hydrochlorothiazide are distinctly different, in a controversial phenomenon termed poly*a*morphism.

## Graphical abstract

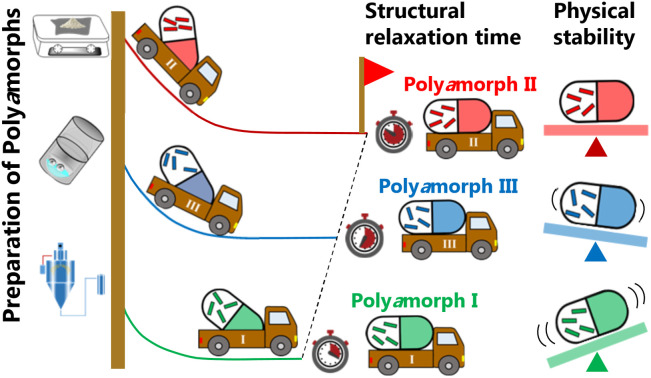

The most obvious criterion between a medicinal or poisonous effect of any substance is its dose. Significantly lowering the dose of a drug in a medicinal formulation is the key to reducing the toxic side effects currently affecting many oral formulations prepared with crystalline drugs. Good bioavailability of drugs in oral formulations requires them to present good solubility in water as a surrogate measure of their solubility in aqueous body fluids. However, the solubility of crystalline materials is limited by their intermolecular long-range order, where high energy is required to disrupt these interactions.

The research community has been investigating a problematic but effective solution. Drug solubility is greatly improved by replacing poorly water-soluble crystalline forms for their amorphous forms. Unlike crystalline solids (Fig. 1, ①), amorphous solids lack long-range order (Fig. 1, ④). Amorphous solids are unambiguously identified using differential scanning calorimetry (DSC) on the emergence of the glass transition temperature *T*_g_, as illustrated in Fig. 1. Only below the *T*_g_ does the solid stay in the amorphous state (also named the glass state). Above the *T*_g_, the solid becomes rubbery (named a supercooled liquid/melt) and can even crystallise. Crystalline solids can be rendered amorphous by processes such as spray drying (SD), quench cooling (QC) and ball milling (BM). BM is the most commonly employed technique to prepare amorphous forms, but for drugs, only those with a *T*_g_ well above the ambient temperature can become amorphized by BM.^1^ If a drug compound has a lower *T*_g_ than the ambient temperature, it will become crystalline upon milling.^2^ Since BM is typically performed at ambient temperatures, and the temperature does not rise above 35 °C in long milling,^2^ not all drugs can be amorphized *via* BM. Alternative techniques for amorphization, including SD or QC,^3–5^ are currently the topic of research by Rades *et al.*, as well as various analytical methods for the characterisation of the amorphous materials.^6–8^

**Fig. 1:**
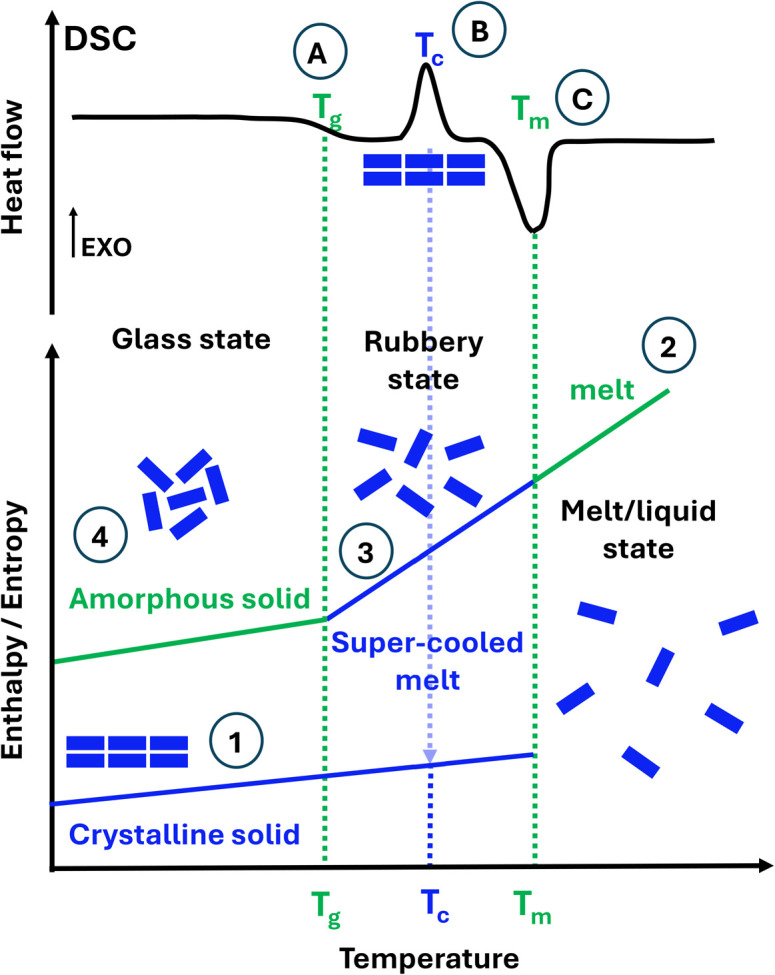
Bottom: conceptual illustration of the energetic and organisational behaviour of ① crystalline and ④ amorphous solids with respect to the increase in temperature. Starting from the ① crystalline solid, the ② melt/liquid state is obtained upon heating. From this ② melt/liquid state, on cooling down, a rubbery state is achieved in the ③ super-cooled melt, leading on further cooling to the ④ amorphous solid. Top: differential scanning calorimetry (DSC) profile for how a controllable increase or decrease in temperature produces (A) the glass transition temperature (*T*_g_), (B) an exothermic crystallisation temperature event (*T*_c_), and (C) an endothermic melt temperature event (*T*_m_).

So, why are amorphous drugs problematic? As illustrated in Fig. 1, amorphous materials ④ have higher energy than their crystalline counterparts ①. This results in poor physical stability over time as they may become crystalline, therefore offering too short a shelf-life for commercialisation. The determination of structural relaxation of a freshly prepared amorphous material *via* isothermal microcalorimetry can be considered a surrogate indicator of its physical stability against crystallisation. All amorphous materials will relax at some point, even if they do not crystallise on the experimental time scale.

What is blocking the rapid development of making amorphous drugs a pharmaceutical reality is the lack of suitable analytical tools to characterise their molecular-level organization. X-ray diffraction, so helpful for determining the molecular-level organisation of crystalline materials, is unsuitable in its conventional way for amorphous materials. Powder X-ray diffraction (PXRD) scans are featureless for amorphous compounds, in comparison to crystalline species, which display distinct peaks (see Fig. 2a). While solid-state NMR can provide structural information on amorphous materials, conducting these experiments is very demanding. On the other hand, pair distribution functions (PDFs),^10^ and molecular dynamics (MD) simulations,^11^ are being explored to obtain some, though limited, understanding of the molecular-level organization of amorphous materials.

**Fig. 2:**
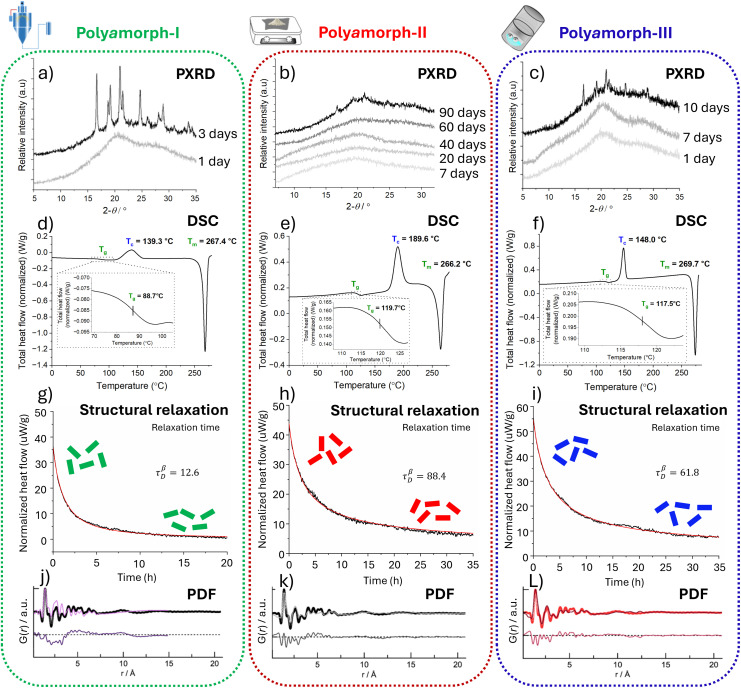
Analytical data from powder X-ray diffraction (PXRD), differential scanning calorimetry (DSC), structural relaxation and pair distribution functions (PDFs); left column (a, d, g & j): poly*a*morph-I obtained by SD; middle column (b, e, h & k): poly*a*morph-II obtained by QC; and right column (c, f, i & l): poly*a*morph-III obtained by BM. Top row (a–c): superimposed PXRD scans of HCT poly*a*morph stability study. Poly*a*morph-II (b) has the highest physical stability while poly*a*morph-I (a) has the lowest, becoming crystalline 3 days after preparation. Second row down (d–f): DSC studies for all 3 poly*a*morphs: I, II and III are different as they present different *T*_g_ and *T*_c_ values. Third row down (g–i): the structural relaxation data showing significant differences between all 3 poly*a*morphs. Poly*a*morph-II (h) with the slowest relaxation *τ*^β^_D_ = 88.4 will have the highest physical stability. Bottom row (j–l): PDF data (probability (*G*) of finding a pair of atoms at a given *r* distance), unambiguously demonstrating that for any of the 3 poly*a*morphs, there is no long-range order (range of >7 Å) corresponding to the intermolecular interactions. It also excludes the presence of crystalline and nanocrystalline material. The originals of all the graphs (ref. 9) were supplied by courtesy of Inês C. B. Martins to modify them as felt fit for the preparation of this figure.

Several reports show that amorphous drugs, obtained using different preparation methods, display distinct physicochemical and thermal properties.^12^ This anecdotal data motivated Martins and Rades, *et al.* to work on dispelling the belief that poly*a*morphs of organic compounds are a fiction.^9^ Poly*a*morphism is defined as the formation of two or more amorphous forms of a single compound, separated by a phase transition.^13^

For the first time, Martins and Rades, *et al.* performed a comprehensive and well-structured proof-of-concept investigation to demonstrate the unequivocal existence of 3 poly*a*morphs (I, II and III) of hydrochlorothiazide (HCT).^9^ Starting from crystalline HCT, poly*a*morph-I was prepared by SD, poly*a*morph-II by QC and poly*a*morph-III by BM. Interestingly, while poly*a*morph-I and III can be transformed to poly*a*morph-II by QC, it is not possible to transform poly*a*morph-II to poly*a*morph-I by SD and to poly*a*morph-III by BM.

Fig. 2 summarizes the 4 analytical techniques applied to characterise the 3 poly*a*morphs I, II and III.^9^ DSC supplies the characterising *T*_g_ and isothermal microcalorimetric analysis supplies the structural relaxation. These parameters unmistakably differentiate between the 3 HCT poly*a*morphs. Poly*a*morph-I with a *T*_g_ of 88 °C, poly*a*morph-II with a *T*_g_ of 119 °C, and poly*a*morph-III with a *T*_g_ of 117.5 °C, have very different glass transition temperatures. A further confirmation that poly*a*morph-II and -III are different is that, on further heating, poly*a*morph-II reaches a crystallisation temperature (*T*_c_) of 189 °C while poly*a*morph-III only reaches a *T*_c_ of 148 °C.^9^

The differences in relaxation between the 3 poly*a*morphs are striking, as shown in Fig. 2. Poly*a*morph-II takes the longest to relax, making it the most physically stable HCT poly*a*morph.^9^ While PXRD and PDF do not discriminate between the different poly*a*morphs, as shown in Fig. 2, they are complementary analytical techniques that support that all 3 HCT poly*a*morphs are fully amorphous. PXRD can confirm the absence of crystalline material, but it cannot confirm the absence of nanocrystals. However, PDF analysis performed at high energy synchrotron facilities can confirm the total absence of crystals and even nanocrystals. PDF analysis confirms that there are no differences between the intramolecular and first-neighbour intermolecular interactions of the 3 HCT poly*a*morphs. The difference must therefore lie at the long-range scale.

To make poly*a*morphism a reality, the scientific community needs to develop new analytical techniques, most likely combined with computational methods, to accurately determine the molecular-level organization (in other words, the structure) of amorphous materials. In summary, it matters how the amorphous forms are prepared, as some poly*a*morphs will have better physicochemical properties than others. The immediate benefit of in-depth studies of poly*a*morphism is obvious: discovering drug poly*a*morphs with improved solubility and physical stability. The future of poly*a*morphism should be bright with safer and more affordable oral formulations in the marketplace.

## Author contributions

Article written by Ana M. Belenguer.

## Conflicts of interest

There are no conflicts to declare.
